# Strengthening Mechanisms in CoCrFeNiX_0.4_ (Al, Nb, Ta) High Entropy Alloys Fabricated by Powder Plasma Arc Additive Manufacturing

**DOI:** 10.3390/nano11030721

**Published:** 2021-03-12

**Authors:** Yupeng Zhang, Qingkai Shen, Xizhang Chen, Subramanian Jayalakshmi, Ramachandra Arvind Singh, Sergey Konovalov, Vladislav B. Deev, Evgeny S. Prusov

**Affiliations:** 1 College of Mechanical and Electrical Engineering, Wenzhou University, Wenzhou 325035, China; z1275377837@gmail.com (Y.Z.); k932034812@gmail.com (Q.S.); jayalakshmi.subramanian@gmail.com (S.J.); arvindsingh.r@gmail.com (R.A.S.); 2 Department of Metals Technology and Aviation Materials, Samara National Research University, 443086 Samara, Russia; ksv@ssau.ru; 3 Department of Metal Forming, National University of Science and Technology “MISiS”, 119049 Moscow, Russia; deev.vb@mail.ru; 4 Department of Functional and Constructional Materials Technology, Vladimir State University, 600000 Vladimir, Russia; eprusov@mail.ru

**Keywords:** high entropy alloy, nanoindentation, strengthening mechanism, EBSD, powder plasma arc additive manufacturing

## Abstract

In high entropy alloys (HEAs), the addition of large-size atoms results in lattice distortion and further leads to solid solution strengthening or precipitation strengthening. However, the relationship between atomic radius, solid solution strengthening and precipitation strengthening has not been discerned yet. In this work, CoCrFeNiX_0.4_ (X = Al, Nb, Ta, with an equi-atomic radius) HEAs were prepared by powder plasma arc additive manufacturing (PPA-AM) and evaluated for their mechanical properties. Compression and nano-indentation hardness tests showed that the HEA with Ta showed the best properties. The influence of atomic radius and solid solubility on solid solution strengthening was investigated and the main strengthening mechanism that determines the mechanical properties of the developed HEAs was analyzed. The results showed that (i) the CoCrFeNiAl_0.4_ alloy did not show any solid solution strengthening effect and that a clear relation between solid solution strengthening and atomic size was not observed; (ii) in both CoCrFeNiTa_0.4_ and CoCrFeNiNb_0.4_ HEAs, precipitation strengthening and grain boundary strengthening effects are observed, wherein the difference in mechanical properties between both the alloys can be mainly attributed to the formation of fine eutectic structure in CoCrFeNiTa_0.4_; and (iii) from the microstructural analyses, it was identified that, in the CoCrFeNiTa_0.4_ HEA, the location containing a fine eutectic structure is accompanied by the formation of low-angle grain boundaries (LAGBs), which is also the region where deformed grains gather, giving rise to improved mechanical strengthening.

## 1. Introduction

Since the introduction of high entropy alloys (HEAs) [1,2], researchers have continuously explored the factors that affect the mechanical properties of HEAs [3,4,5,6,7,8,9,10]. The solid solution phase is the main component of the high entropy alloy, and different phases (such as FCC, BCC, and HCP) have a great influence on the mechanical properties of HEAs. In recent research [11,12,13], solid solution strengthening has been more used in CoCrFeNi-based HEAs added with a large atomic radius to explain the improvement of alloy properties. However, the researchers did not give specific proof of solid solution strengthening. The strengthening effect of the precipitated phase is significant [14,15,16,17], and the mechanical properties of the alloy will be significantly enhanced, as the volume fraction of the precipitated phase increases. However, the difference in strengthening effects of various phases is worth studying to guide the design of HEAs. For grain boundary strengthening, researchers often use rolling, annealing, and other processes to refine the grains of the alloys, and use more grain boundaries to hinder the movement of dislocations during the deformation process, thereby increasing the strength of the alloy [18,19,20]. As we all know, there are many factors that affect the properties of the alloy in the process of alloy transition from liquid to solid. Whether solid solution strengthening is caused by the addition of a large atomic radius and how other strengthening mechanisms (precipitation strengthening, grain boundary strengthening, etc.) affect the mechanical properties should be clarified. Wu et al. [21] has made it clear that the mechanical properties of the alloy can be accumulated by the effects of the individual strengthening mechanisms (Equation (1)). This provides theoretical support for proving the relationship between solid solution strengthening and atomic radius in this work.

(1) $$\sigma_{y}=\sigma_{fr}+\Delta \sigma_{pi}+\Delta \sigma_{ss}+\Delta \sigma_{ppt}+\Delta \sigma_{gb}$$

Here, $\sigma_{fr}$ is the frictional stress; $\Delta \sigma_{pi}$ is the various incremental strengthening contributions; $\Delta \sigma_{ss}$ is the solid solution strengthening; $\Delta \sigma_{ppt}$ is the precipitation strengthening; and $\Delta \sigma_{gb}$ is the grain boundary strengthening.

Most of the existing HEAs are prepared in vacuum arc melting furnaces [1,12], which greatly limits the size and shape of the HEAs. The researchers used laser deposition additive manufacturing to prepare the HEA to overcome this limitation [11,22]. However, high-power laser equipment is expensive, and a low deposition efficiency is not conducive to large-scale applications in the market. The powder plasma arc additive manufacturing system has the advantages of low price, high deposition efficiency, and low surface roughness [23,24]. It has greater potential to be used in future industrial production.

In this work, the widely researched CoCrFeNi alloy was selected. Elements (Al, Ta, Nb = 143 p.m. [9]) with equal atomic radius were added to verify the relationship between solid solution strengthening and atomic size. CoCrFeNiX_0.4_ (X = Al, Ta, Nb) HEAs with equal entropy were prepared by powder plasma arc additive manufacturing (PPA-AM) and evaluated for their mechanical properties. The following were investigated in order to identify the strengthening mechanism in the developed HEAs: (i) phase crystal structure and texture; (ii) the nanohardness of the FCC and precipitated phase; and (iii) the relationship between eutectic structure, low angle grain boundaries, and deformed grains.

## 2. Materials and Methods

The purity of each metal powder (200 mesh size) was above 99.5% and they were mixed in a high-energy ball mill (YXQM-12L, MITR Ltd., Changsha, China) with 3 times the mass of the Al_2_O_3_ grinding balls for 12 h to ensure powder homogenization. The powder was dried in vacuum for two hours. Prior to deposition on the steel substrate, impurities were removed by cleaning the surface with acetone and ultrasonic treatments. Bulk HEAs with a nominal composition of CoCrFeNiX_0.4_ (X = Al, Nb, Ta) were prepared by the powder plasma arc additive manufacturing (PPA-AM, DML-VO3AD, Duomu Company, Shanghai, China). The deposition current was 110 A (affect sample roughness); travel speed was 400 mm/min (affect sample roughness); pulse period was 63 us; pulse interval was 10 us; shielding gas was 9 L/min; powder feeding gas was 4 L/min; powder feed velocity was 35 round/min (affect sample roughness); and more deposition parameters can be found in [23]. Under the shield gas of an Ar atmosphere, the torch with coaxial powder feeding was stacked layer by layer.

The microstructure of the printed HEA specimens was observed using a scanning electron microscope (SEM, Zeiss Sigma 300, Analytik Jena AG, Jena, Germany) and transmission electron microscope (TEM-JEM-2100, Jeol, Tokyo, Japan). The grain size and orientation were investigated by Oxford NordlysMax2 electron backscatter diffraction (EBSD, TESCAN China, Ltd., Brno, Czech Republic). The nanohardness of each phase was measured by a nanoindenter (G200 Keysight, Santa Rosa, CA, USA), with point spacing of 100 μm, and eleven points were selected under a load of 4.2 mN for 30 s. In terms of microstructure observation (SEM), the sample was gradually ground, and then polished and etched using aqua regia (CoCrFeNi and CoCrFeNiAl_0.4_) and hydrofluoric acid (CoCrFeNiTa_0.4_ and CoCrFeNiNb_0.4_). Compression tests were carried out in a universal testing machine (Hualong WDW -100, Shanghai, China) at a strain rate of 10^−3^ s^−1^; three tests were conducted and the average values were plotted.

## 3. Results

**Figure 1:**
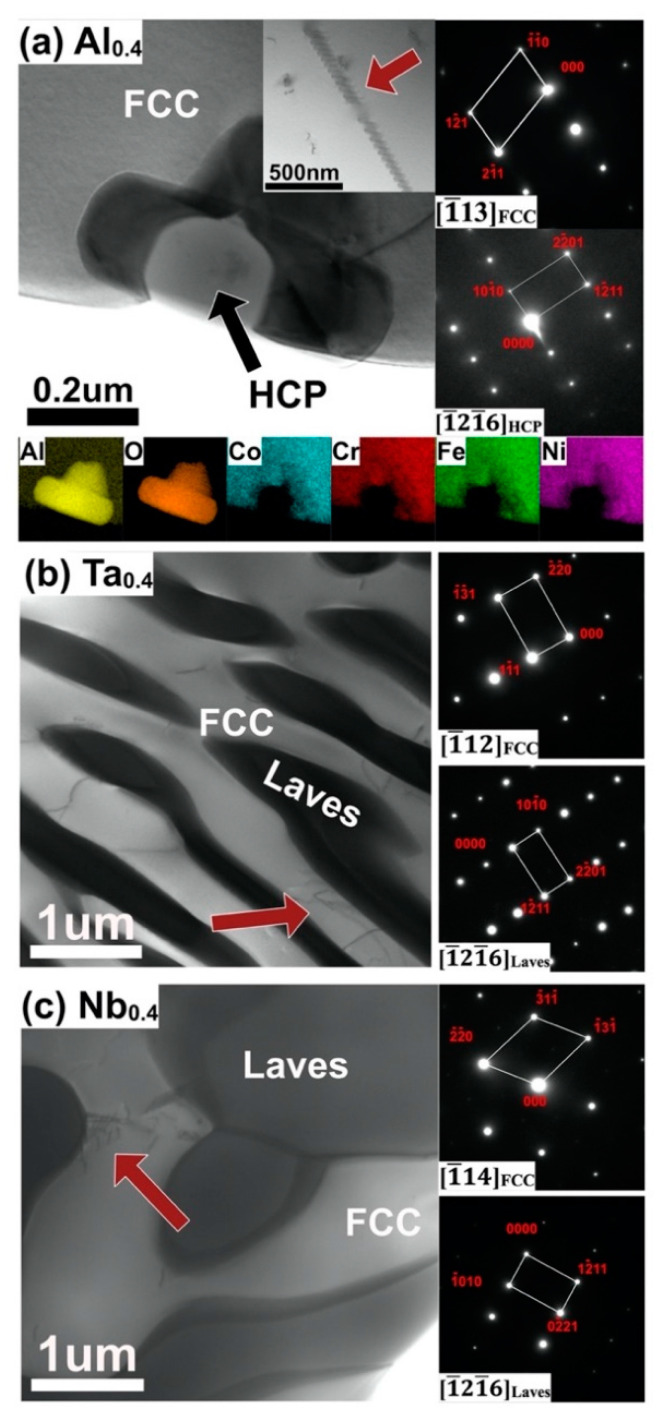
TEM micrographs, with the illustration of the selected area diffraction pattern (SADP) in (**a**) CoCrFeNiAl_0.4_, (**b**) CoCrFeNiTa_0.4_, and (**c**) CoCrFeNiNb_0.4_ high entropy alloys (HEAs), prepared by powder plasma arc additive manufacturing (PPA-AM).

The structure and crystal type of the CoCrFeNiX_0.4_ (Al, Ta, Nb) HEAs produced by powder plasma arc additive manufacturing (PPA-AM) [23] are shown in the TEM images in Figure 1. The selected area diffraction pattern (SADP) of the white area is $\left[\bar{1}13\right]$_FCC_ phase, as shown in Figure 1a. In addition to the well-known BCC phase [5,25,26], alumina with an HCP structure was also detected, as shown in Figure 1a. This may be caused by oxidation in the PPA-AM process. In Ta_0.4_ and Nb_0.4_ alloys, the white phases of $\left[\bar{1}12\right]$_FCC_ and $\left[\bar{1}14\right]$_FCC_ were detected, respectively; also observed were the $\left[\bar{1}2\bar{1}6\right]$_Laves_ phase rich in Co-Ta (as shown in Figure 1b) and the $\left[\bar{1}2\bar{1}6\right]$_Laves_ phase rich in Fe-Nb (as shown in Figure 1c). This result is consistent with previous XRD [23] and is also similar to HEAs produced by vacuum arc melting and direct laser deposition AM [5,14,17,27], except for the alumina with an HCP structure in CoCrFeNiAl_0.4_. Since no oxides were detected in the Ta_0.4_ and Nb_0.4_ alloys, the influence of the Al_2_O_3_ grinding balls and TEM sample preparation were excluded. The sporadic presence of oxides detected in the CoCrFeNiAl_0.4_ alloy is probably due to the ease of oxidation of aluminum, indicating that the vacuum was not sufficient to prevent oxidation given the high temperature involved in the deposition process. In addition, the dislocations in the FCC phase are shown by the red arrows in Figure 1. which may be related to the strengthening mechanism of the three HEAs. This point will be analyzed below.

**Figure 2:**
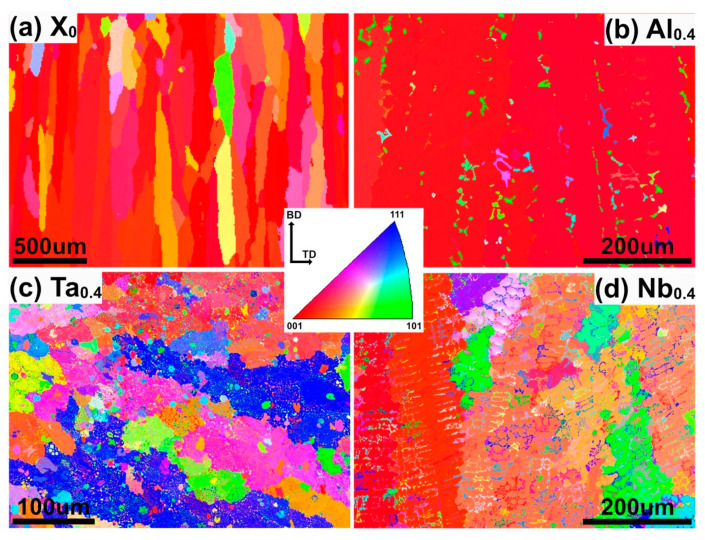
IPF of (**a**) CoCrFeNi, (**b**) CoCrFeNiAl_0.4_, (**c**) CoCrFeNiTa_0.4_, and (**d**) CoCrFeNiNb_0.4_ HEAs, prepared by PPA-AM.

Figure 2 shows the EBSD scan of the CoCrFeNiX_0.4_ HEAs. The FCC phase tends to grow along the <001> direction during the selective laser melting AM process [28]. Similar to that reported in [27], the HEAs with X_0_ and Al_0.4_ deposited by PPA-AM also are long elongated and oriented in <001>. However, the texture of FCC changes in Ta_0.4_ and Nb_0.4_ HEAs due to the increase in the precipitated phase volume [13], as shown in Figure 2c,d. Ta_0.4_ and Nb_0.4_ alloys both appear to have short grains, which is oriented in a different direction (<001>, <111>, <101>); i.e., a more randomized structure. In particular, the Ta_0.4_ alloy has a completely random grain orientation. The grain size of CoCrFeNi has been refined, as seen from the IPF diagram of the Ta_0.4_ and Nb_0.4_ alloy.

**Figure 3:**
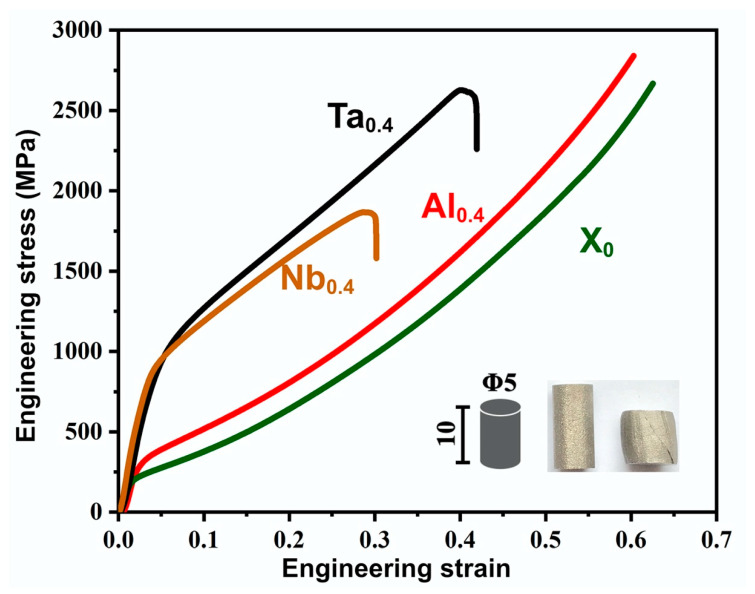
Compressive test result of the CoCrFeNiX_0.4_ HEA prepared by PPA-AM.

The compression stress–strain curves are shown in Figure 3. The CoCrFeNi alloy has the lowest yield strength, and the Al addition slightly increases the strength of CoCrFeNi in the compression test. Both have no brittle fracture due to good plasticity (note that the tests were stopped at 0.6 strain). Comparatively, for a particular strain value, the Ta and Nb alloys exhibit a high yield and fracture strengths. Among them, the fracture strength of the Ta_0.4_ alloy is better than the traditional vacuum arc melting process [16,17,29]. Since the Ta_0.4_ and Nb_0.4_ alloys have the same precipitation phase [14,16,23], the difference in strength may be related to the formation of a fine eutectic structure in the Ta_0.4_ alloy.

## 4. Discussion

Solid solution strengthening, precipitation strengthening, and grain boundary strengthening play different roles in determining the mechanical properties of HEAs. In most research works, solid solution strengthening caused by lattice distortion was identified to be the dominant mechanism to explain the difference in mechanical properties when an element with a large atomic radius was added to the CoCrFeNi alloy [11,12,13,30]. Precipitation strengthening is common in dual-phase HEAs, but the number of reports in the literature that explain the relationship between the volume fraction of the precipitated phase and the nanohardness of the phase to that of the macroscopic mechanical properties are relatively less [14,15,17,30]. In addition, the grain boundary strengthening mechanism was also used to explain the difference in mechanical properties by the Hall–Petch equation [19,20,31], wherein the smaller the grain size, the higher the strength of the alloy. Therefore, the influence of grain boundary size on the mechanical properties of the alloy has been qualitatively analyzed.

There also is an inseparable relationship between dislocation strengthening and grain or interphase boundary [32]. It has been reported recently that the microstructure difference between the top and bottom layers in AM-deposited HEAs was caused by the repeated heat flow over the bottom printed layer [23]. In the AM process, the high cooling rate will inevitably cause the accumulation of internal stress in the material, which will further lead to the slip transmission of dislocation [33,34]. The contribution from various strengthening mechanisms on the mechanical properties of the developed CoCrFeNiX_0.4_ HEAs are addressed in the following section.

### 4.1. Solid Solution Strengthening

**Figure 4:**
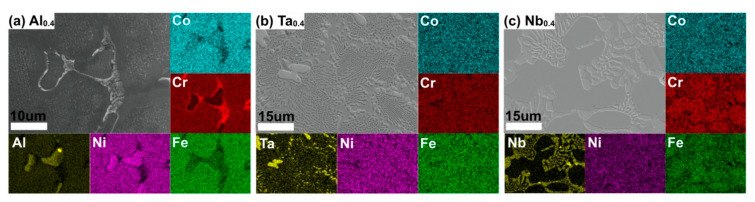
The EDS Mapping of the (**a**) CoCrFeNiAl_0.4_, (**b**) CoCrFeNiTa_0.4_, and (**c**) CoCrFeNiNb_0.4_ HEAs prepared by PPA-AM.

Large-size atoms dissolve in the matrix, resulting in solid solution strengthening due to lattice distortion [12,30]. In order to understand the degree of enrichment of atoms in each phase, the EDS mapping of CoCrFeNiX_0.4_ is shown in Figure 4. It can be seen from that X (Al, Ta, Nb) has indeed dissolved in the FCC phase.

From the composition difference of X (Al, Ta, Nb) in the FCC phase [23], the content of Al (10.97 at%) is higher than that of Ta (2.36 at%) and Nb (2.68 at%). The relative degree of lattice distortion produced by the FCC phase can be calculated by δ [35], which is used to characterize the atomic size mismatch. Considering the FCC phase is regarded as a single HEA, then the atomic size mismatch of the FCC phase in CoCrFeNiX_0.4_ can be calculated according to the following Equation (2) [35,36]:

(2) $$\delta =\sqrt{\overset{N}{\underset{i=1}{\sum }}C_{i}{\left(1-\frac{r_{i}}{\sum_{i=1}^{N}c_{i}r_{i}}\right)}^{2}}$$

Here, N is the number of components in HEAs, $C_{i}$ is the atomic percentage of the *i*-th element in FCC phase, $r_{i}$ is the atomic radii of i-th element, and $\bar{r}=\sum_{i=1}^{N}c_{i}r_{i}$ is the average atomic radii in FCC phase. The atomic size mismatch (*δ*) of the FCC phase in CoCrFeNiX_0.4_ (Al, Ta, Nb) are 4.52%, 2.24%, and 2.38%, respectively. It is evident that the large solid solubility of the Al atom in the FCC phase leads to a larger atomic size mismatch, and vice versa. However, the effect of greater solid solubility of Al in the FCC phase contributing towards greater strengthening effect has to be understood.

**Figure 5:**
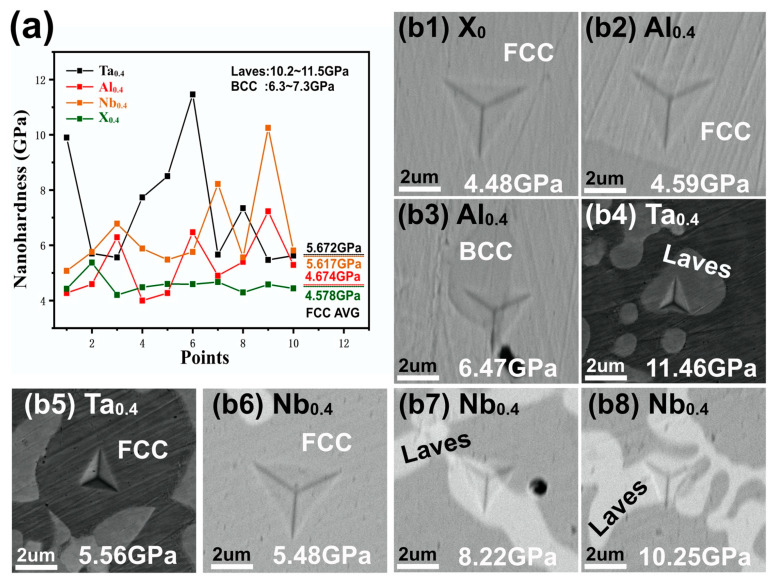
(**a**) The nanohardness distribution and (**b**) nanoindentation morphology of (**b1**) FCC in CoCrFeNi, (**b2**) FCC in CoCrFeNiAl_0.4_, (**b3**) BCC in CoCrFeNiAl_0.4_, (**b4**) Laves in CoCrFeNiTa_0.4_, (**b5**) FCC in CoCrFeNiTa_0.4_, (**b6**) FCC in CoCrFeNiNb_0.4_, (**b7**,**b8**) Laves in CoCrFeNiNb_0.4_.

Figure 5 shows (a) the distribution of nanohardness and (b) the SEM morphology of the nanoindentation. The nanohardness of the FCC phase is about 4.578 GPa in the CoCrFeNi HEA, and the morphology is as shown in Figure 5(b1). Figure 5(b2) shows the nanoindentation morphology of the FCC phase in CoCrFeNiAl_0.4_. Although Al has a higher solid solubility in CoCrFeNi, the average hardness (4.674 GPa) of FCC in CoCrFeNiAl_0.4_ is equal to that of CoCrFeNi. Interestingly, Figure 5(b5,b6) are the nanoindentation morphology of FCC in Ta_0.4_ and Nb_0.4_ HEAs, and their average nanoindentation hardness is about 5.672 and 5.617 GPa, as shown in Figure 5a. From the nanohardness of FCC phase in CoCrFeNiX_0.4_, it can be seen that the high atomic solid solubility does not necessarily cause solid solution strengthening. Moreover, the solid solution strengthening effect is not directly related to the atomic size.

### 4.2. Precipitation Strengthening

**Figure 6:**
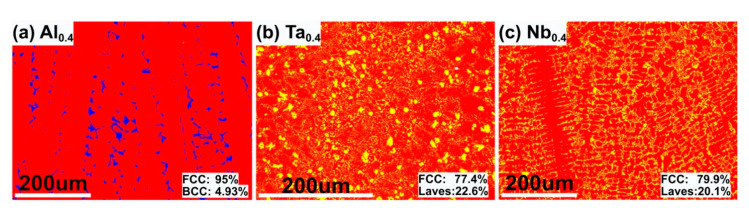
The phase distribution of the (**a**) CoCrFeNiAl_0.4_, (**b**) CoCrFeNiTa_0.4_, and (**c**) CoCrFeNiNb_0.4_ HEAs prepared by PPA-AM.

Precipitation strengthening is a dominant strengthening mechanism in HEA with two or three phases. However, the effect of phase nanohardness has often been ignored while analyzing the effect of the precipitated phase volume fraction on macro-mechanical properties [14,15,16,17,37]. It can be seen from Figure 5 that the hardness of the precipitated phases is higher than that of the FCC phase. Figure 6 shows the volume fraction of the precipitated phase. The nanohardness and volume fraction of the BCC phase are both at a relatively low level. This may be the main reason for the lower strength of the Al_0.4_ HEA.

The hardness of the Laves phase is between 10.2 and 11.5 GPa, as shown in Figure 5. With the increase in Laves phase in the indentation, the nanohardness will continue to increase according to Figure 5(b7,b8). Therefore, the Ta_0.4_ and Nb_0.4_ HEAs are likely to have the same hardness value. Furthermore, the volume fraction of the Laves phase in the Ta_0.4_ (22.6%) alloy is slightly larger than that in the Nb_0.4_ (20.1%) alloy. Therefore, the effect of precipitation strengthening in the Ta_0.4_ alloy is slightly stronger than that of the Nb_0.4_ alloy.

### 4.3. Grain Boundary Strengthening

**Figure 7:**
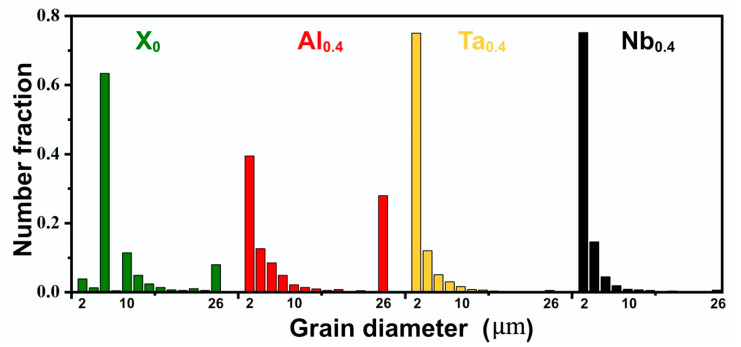
Grain size distribution of the CoCrFeNiX_0.4_ (X = Al, Ta, Nb) HEAs prepared by PPA-AM.

From the above discussions, it could be seen that the difference in mechanical properties of the Ta_0.4_ and Nb_0.4_ HEAs could not be explained based on the contribution from the precipitation strengthening mechanism alone. Grain boundary strengthening, as a strengthening mechanism in metal materials, is often used to increase the strength of materials through rolling or heat treatment. In the present work, a comparison of grain diameter of CoCrFeNiX_0.4_ alloys (Figure 7) has been made in order to understand the difference in mechanical properties. It is evident that a large number of large-sized grains are found in X_0_ and Al_0.4_ alloys, especially grains above 26 μm. However, in Ta_0.4_ and Nb_0.4_ alloys the grain sizes are similar. Due to the similar grain size distribution, it is impossible to accurately distinguish the effect of grain size on the mechanical properties of the Ta_0.4_ and Nb_0.4_ alloys from the grain size distribution in Figure 7.

**Figure 8:**
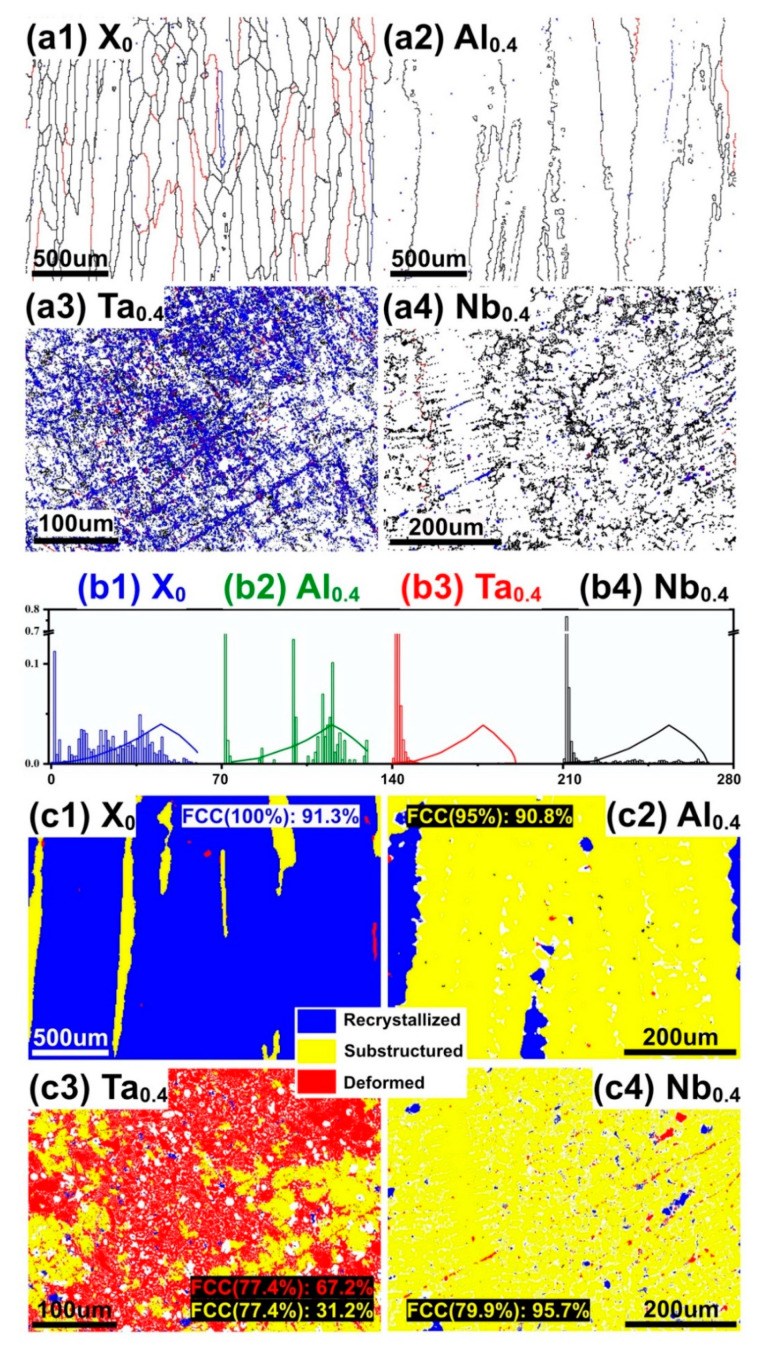
(**a**) Grain boundary of (a1) CoCrFeNi, (a2) CoCrFeNiAl_0.4_, (a3) CoCrFeNiTa_0.4_, (a4) CoCrFeNiNb_0.4_, (**b**) misorientation angle distribution of (b1) CoCrFeNi, (b2) CoCrFeNiAl_0.4_, (b3) CoCrFeNiTa_0.4_, (b4) CoCrFeNiNb_0.4_, and (**c**) DefRex map of (c1) CoCrFeNi, (c2) CoCrFeNiAl_0.4_, (c3) CoCrFeNiTa_0.4_, (c4) CoCrFeNiNb_0.4_.

To understand much further, during the AM process, the bottom layer of sample will undergo repeated heat flow. In the process of the material changing from liquid to solid, thermal shrinkage and volume change will occur inside the printed sample due to the dramatic change in temperature [38]. The stress generated in this process will cause the accumulation and rearrangement of dislocations [39]. The occurrence of recrystallization may also be the cause of changes in the structure between the top and bottom layers [23]. In grain boundary engineering, the influence of dislocation density on mechanical properties can be qualitatively judged by the angle of the grain boundary and recrystallization. Therefore, the grain boundary, orientation angle distribution, and recrystallization of CoCrFeNiX_0.4_ were studied, as shown in Figure 8. Grain boundaries with different misorientations, such as >2°, >5°, >15°, are indicated by blue, red, and black, respectively, in Figure 8. The misorientations of the grain boundaries higher than 15° are defined as high-angle grain boundaries (HAGBs) and those <15° are called as low-angle grain boundaries [40]. According to the EBSD results in Figure 8a,b, the misorientations of the grain boundaries show a significant difference with the change of elements, although the Ta_0.4_ alloy has similar macro-mechanical properties and nanohardness than the Nb_0.4_ alloy. The DefRex diagram from EBSD shows the crystallization status, which reflects the existence of dislocations to a certain extent [41], as shown in Figure 8c. Deformed grains (marked as red) indicate that the interior is full of dislocations; the formation of the substructure (yellow) needs to consume and absorb some dislocations, and there is basically no dislocation formation inside the recrystallized (blue). The blue text in Figure 8(c1) indicates that the FCC phase occupies 100% of the CoCrFeNi alloy, and the recrystallization ratio reaches 91.3% in the FCC phase. Similarly, it can be seen that the Ta_0.4_ alloy contains a large number of deformed grains (67.2%) and recrystallized grains (31.2%). Combining Figure 6b and Figure 8(a3,c3), it can be seen that the eutectic structure is accompanied by the formation of a large number of LAGBs, and the aggregation of LAGBs represents deformed grains. While alloys with a high yield strength are accompanied by the formation of denser LAGBs [39], the phenomenon of crack aggregation was reported to occur at HAGBs [42]. Dislocations are often concentrated in deformed grains, which will inhibit the plastic deformation of the alloy and increase the strength of the alloy. It can be seen in Figure 8(a3) that the existence of a fine eutectic structure is accompanied by the production of LAGBs, which contribute towards an increase in the mechanical properties of the Ta_0.4_ alloy when compared to that of the Nb_0.4_ alloy. From the EBSD analysis, it is evident that dislocation strengthening manifests in the Ta_0.4_ alloy and is the key reason for its higher values of mechanical properties when compared to that of the Nb_0.4_ alloy.

## 5. Conclusions

In the present work, Al, Ta, and Nb of equal atomic radius were added to a CoCrFeNi HEA and manufactured by PPA-AM. The microstructural evolution and compression properties of the deposited alloys were investigated. The strengthening mechanisms, namely, solid solution strengthening, precipitation strengthening, grain boundary strengthening, and dislocation strengthening, were analyzed. Following are the conclusions that could be drawn from the study.

1. The mechanical properties of the developed alloys were in the order CoCrFeNiTa_0.4_ > CoCrFeNiNb_0.4_ > CoCrFeNiAl_0.4_ > CoCrFeNi alloy;
2. A direct relationship between atomic size and solid solubility with solid solution strengthening was not observed, and the solid solution strengthening effect was not seen in the CoCrFeNiAl_0.4_ alloy;
3. The effect of precipitation strengthening was much higher than that of solid solution strengthening, wherein a similar contribution to strengthening exist due to the precipitation strengthening effect in both the CoCrFeNiTa_0.4_ and CoCrFeNiNb_0.4_ alloys;
4. The effect of strengthening due to grain refinement effects were similar in both the CoCrFeNiTa_0.4_ and CoCrFeNiNb_0.4_ alloys, and are better than Al in the CoCrFeNi alloy;
5. The better enhancement in the mechanical properties of CoCrFeNiTa_0.4_ is attributed to the formation of LAGBs and deformed grains that gathered around the fine eutectic structure in CoCrFeNiTa_0.4_.

## Data Availability

Data available in a publicly accessible repository.
